# Comparative Evolution of SLC12 Transporters in Tilapias and Salinity-Responsive Expression in Nile Tilapia

**DOI:** 10.3390/ijms27188124

**Published:** 2026-09-12

**Authors:** Jin-Min Pan, Liu-Yang Li, Meng-Fan Dong, Qian-Hui Chen, Jun-Hong Xia

**Affiliations:** State Key Laboratory of Biocontrol, Institute of Aquatic Economic Animals and Guangdong Provincial Key Laboratory for Aquatic Economic Animals, College of Life Sciences, Sun Yat-Sen University, Guangzhou 510275, China

**Keywords:** *SLC12*, *Oreochromis*, salinity adaptation, osmoregulation, gene family evolution

## Abstract

Tilapia species differ markedly in salinity tolerance, providing a useful system for investigating the molecular basis of osmotic adaptation. The solute carrier family 12 (*SLC12*) encodes cation–chloride cotransporters that play central roles in ion homeostasis and osmoregulation. Here, we compared the SLC12 gene family among Nile tilapia (*Oreochromis niloticus*), Mozambique tilapia (*O. mossambicus*), and blue tilapia (*O. aureus*) using comparative genomic, phylogenetic, protein-structural, and chromosomal analyses. Separately, we re-analyzed a published long-term salinity RNA-seq dataset from GIFT Nile tilapia maintained for 3.5 months at 0, 17, or 27 ppt, focusing on *SLC12* expression in the gill, intestine, and skin. All three tilapia species contained 17 *SLC12* loci with identical subfamily copy numbers and broadly conserved chromosomal distributions. In the Nile tilapia transcriptomic dataset, paralogous *SLC12A2, SLC12A7*, and *SLC12A10* copies displayed distinct tissue- and salinity-dependent expression patterns. *SLC12A8* also showed salinity-responsive expression in Nile tilapia, prompting further comparative sequence and structural analysis. The three SLC12A8 orthologs were broadly similar in sequence and predicted structure, although several species-specific residue differences were observed. These findings reveal overall conservation of the *SLC12* family among tilapias, identify salinity-responsive expression differences among paralogous members in Nile tilapia and interspecific sequence variation in *SLC12A8*, and provide candidate features for further functional investigation.

## 1. Introduction

Environmental salinity directly determines the osmotic gradient between fish body fluids and the surrounding environment. Therefore, maintaining body-fluid osmolality and inorganic ion homeostasis is a fundamental physiological requirement for fish adapting to different aquatic environments. Freshwater teleosts face continuous ion loss and water influx, whereas fish in seawater or high-salinity environments must cope with water loss and the passive entry of ions such as Na^+^ and Cl^−^. To maintain internal homeostasis, fish rely on the coordinated regulation of ion uptake, ion excretion, and water transport by organs including the gills, intestine, and kidneys, with ionocytes in the gill epithelium serving as the principal sites of transepithelial Na^+^ and Cl^−^ transport. Changes in environmental salinity can remodel ionocyte types and the expression and localization of membrane transport proteins, thereby enabling a physiological transition from ion uptake in freshwater to salt secretion at high salinity [1,2,3].

The solute carrier family 12 (*SLC12*) encodes electroneutral cation–chloride cotransporters, which constitute an important membrane transport system for maintaining intracellular and extracellular Na^+^, K^+^, and Cl^−^ homeostasis, cell volume, and transepithelial ion transport. This family includes Na^+^-K^+^-2Cl^−^ cotransporters (*NKCCs*), Na^+^-Cl^−^ cotransporters (*NCCs)*, K^+^-Cl^−^ cotransporters (*KCCs*), and several members whose functions remain relatively poorly understood [4,5]. Among fish, the osmoregulatory functions of NKCCs and NCCs have been studied most extensively. Hiroi et al. [6] obtained full-length cDNAs for *NKCC1a*, *NKCC1b*, *NKCC2*, *NCC* and other transporters from the gills of Mozambique tilapia and demonstrated that NCC is primarily associated with Na^+^ and Cl^−^ uptake under freshwater conditions, whereas *NKCC1a* is closely associated with salt secretion by seawater-type ionocytes. These findings established the basic framework through which classical *SLC12* members mediate salt uptake in freshwater and salt secretion at high salinity in tilapia.

However, the evolutionary composition of the fish *SLC12* family is substantially more complex than the classical *NKCC*/*NCC* binary model. Teleost-specific genome duplication, followed by the retention and loss of duplicated copies, has resulted in the preservation of paralogs in several *SLC12* subfamilies, and different copies may have subsequently diverged in tissue expression, regulatory responses, and physiological functions. Recent re-evaluation of the evolutionary history of *NCC2*/*SLC12A10* has also shown that it is not simply a “fish-specific gene”; patterns of retention and loss across vertebrate lineages are far more complex than previously recognized [7]. Therefore, the functional diversity of the teleost *SLC12* family cannot be fully explained solely on the basis of known functions of mammalian homologs or a few classical fish transporters. In particular, systematic comparisons remain lacking regarding whether poorly characterized members and paralogs generated through duplication have diverged in protein sequence, membrane topology, and environmental responsiveness.

Tilapias of the genus *Oreochromis* have a strong capacity for environmental adaptation, although their salinity tolerance differs among species. Mozambique tilapia (*O. mossambicus*) is renowned for its pronounced euryhalinity and high-salinity tolerance and can maintain ion homeostasis during high-salinity acclimation by increasing the abundance and activity of chloride cells in the branchial and opercular epithelia [8]. Blue tilapia (*O. aureus*) also exhibits relatively high salinity tolerance, whereas the high-salinity tolerance of Nile tilapia (*O. niloticus*) is comparatively limited and offers substantial scope for genetic improvement [9]. Our previous studies have investigated the genetic and molecular basis of salinity tolerance in Nile tilapia using genetic mapping and multi-omics approaches. QTL-seq identified a major QTL associated with salinity tolerance [10], and subsequent GWAS and QTL-seq analyses further revealed a cluster of salinity-tolerance QTLs on chrLG18 [11]. Candidate genes and osmoregulatory responses were further investigated through skin transcriptomics and integrated gill transcriptomic and metabolomic analyses under long-term salinity exposure [12,13]. These studies have demonstrated a clear genetic and transcriptional regulatory basis for salinity tolerance in tilapia; however, they have focused primarily on a single species, candidate regions within QTLs, or classical ion-regulatory pathways.

Nile tilapia, Mozambique tilapia, and blue tilapia are therefore closely related species with distinct salinity-tolerance characteristics, providing an ideal comparative system for investigating conservation and divergence of the SLC12 family among related species. Among SLC12 members, SLC12A8 is particularly poorly characterized. Mammalian studies have proposed roles for SLC12A8 in polyamine/amino-acid transport and nicotinamide mononucleotide transport, although the latter interpretation has been challenged [14,15,16]. Thus, the substrate specificity and physiological function of SLC12A8, particularly in teleosts, remain unresolved. SLC12A8 was therefore prioritized for further sequence and structural comparison because it combines single-copy conservation across the three tilapia species, poorly characterized function in teleosts, salinity-responsive expression in Nile tilapia, and interspecific amino-acid variation. In this study, we first compared the SLC12 gene family among Nile tilapia, Mozambique tilapia, and blue tilapia using sequence, phylogenetic, structural, and chromosomal analyses. We then separately analyzed a published long-term salinity transcriptome dataset from GIFT Nile tilapia to characterize tissue- and salinity-dependent expression of SLC12 members. Thus, the genomic analyses represent an interspecific comparison, whereas the expression analysis represents a single-species analysis of Nile tilapia. Within this family-wide framework, candidate members showing notable sequence variation or salinity-responsive expression were further examined through protein-sequence comparison, transmembrane-topology prediction, and three-dimensional structural modeling, with SLC12A8 treated as a candidate for functional investigation rather than as an established salt-ion transporter.

## 2. Results

### 2.1. Identification and Protein Characteristics of SLC12 Family Members in Three Tilapia Species

Seventeen *SLC12* family members were identified in each of Nile tilapia, Mozambique tilapia, and blue tilapia, yielding a total of 51 *SLC12* genes. The three tilapia species shared the same family composition: *SLC12A1*, *SLC12A3*, *SLC12A4*, *SLC12A6*, and *SLC12A8* were each represented by a single copy; *SLC12A2*, *SLC12A5*, and *SLC12A7* each had two copies; and *SLC12A9* and *SLC12A10* each had three copies. Comparison across representative vertebrates showed interspecific differences in the total number of *SLC12* loci and in the copy number of each subfamily; all three tilapia species contained 17 *SLC12* loci (Table 1).

The 51 SLC12 proteins ranged from 757 to 1212 aa in length and from 82.55 to 133.66 kDa in molecular weight, with theoretical isoelectric points (pI) of 5.62–8.16, instability indices of 34.17–44.43, and grand average of hydropathy (GRAVY) values of −0.0342–0.4511 (Table 2). OnSLC12A8, OaSLC12A8, and OmSLC12A8 were 757, 762, and 766 aa long, respectively.

TMHMM 2.0 predicted that all 51 SLC12 proteins contained multiple transmembrane helices (Figure S1). Most proteins were predicted to contain 10–12 helices, whereas a small number contained 9 or 13.

### 2.2. Phylogeny, Conserved Motifs, Domains, and Gene Structures of the SLC12 Family

**Figure 1:**
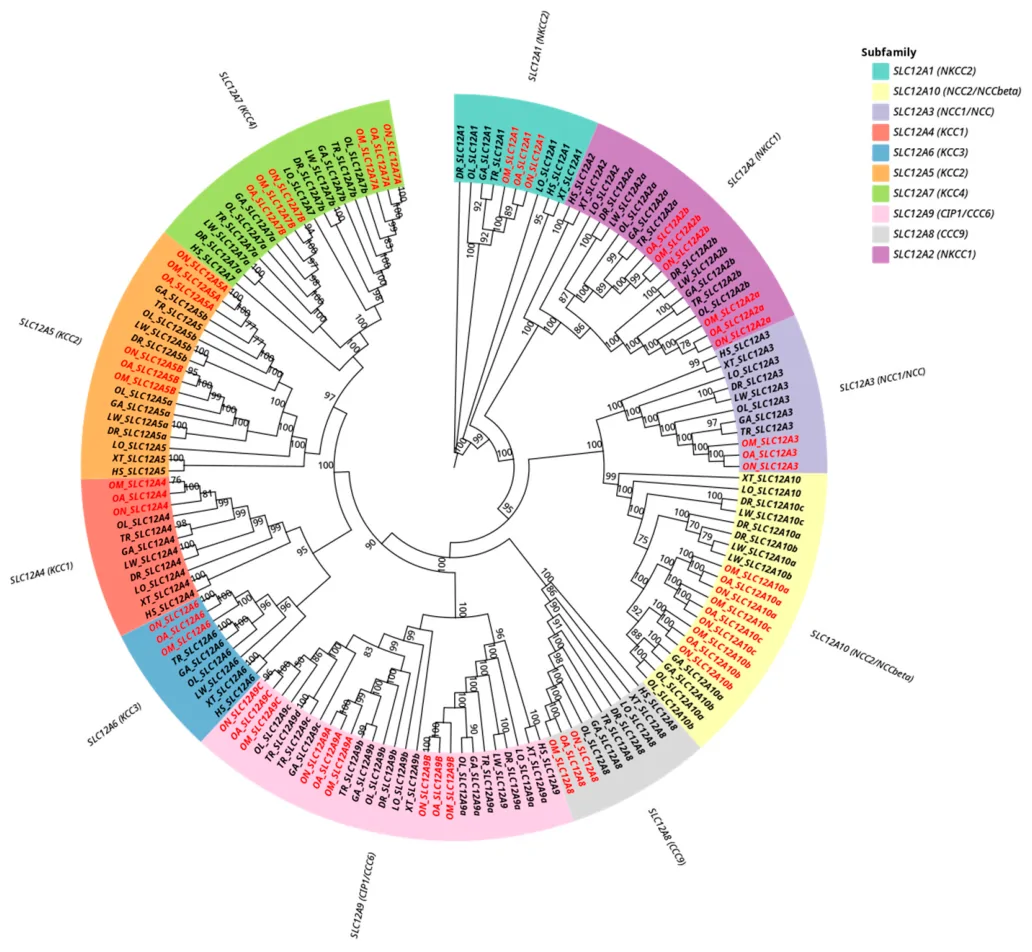
Phylogenetic relationships of SLC12 proteins from representative vertebrates and three tilapia species. Red labels indicate SLC12 members from the three tilapia species; different background colors denote the major SLC12 subfamilies.

Phylogenetic analysis assigned the SLC12 proteins to the major SLC12A1, A2, A3, A4, A5, A6, A7, A8, A9, and A10 subfamilies. Orthologous proteins with the same name from the three tilapia species clustered within their respective subfamilies. SLC12A8 was present as a single copy in each species, and the three orthologs formed a single clade; SLC12A2, SLC12A5, SLC12A7, SLC12A9, and SLC12A10 each contained multiple paralogous copies (Figure 1).

**Figure 2:**
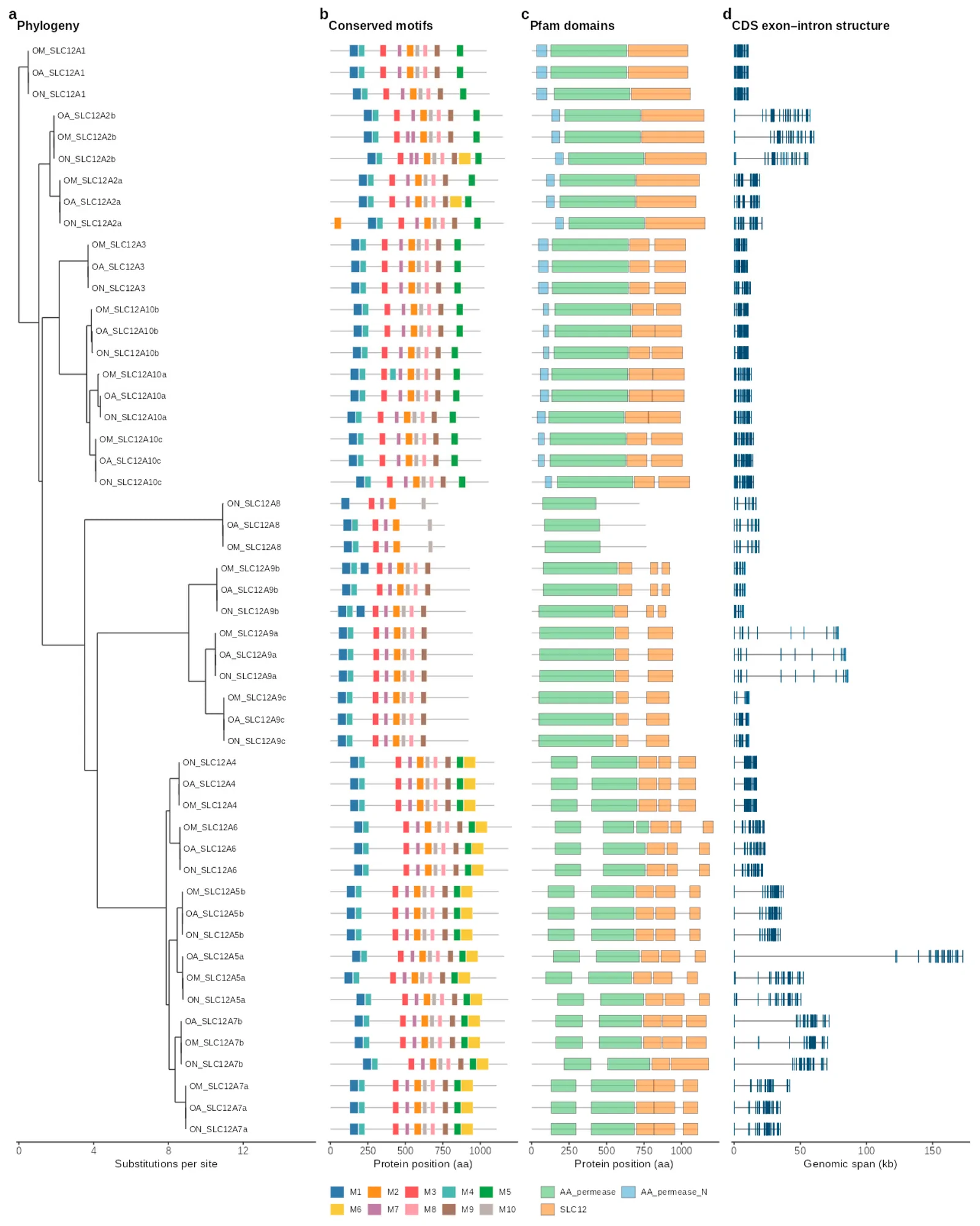
Comparative analysis of the phylogenetic relationships, conserved motifs, Pfam domains, and CDS exon–intron structures of SLC12 family members in three tilapia species. (**a**) Phylogenetic relationships among 51 SLC12 proteins from the three tilapia species. (**b**) Conserved motif composition and distribution along the protein sequences; M1–M10 denote distinct conserved motifs. (**c**) Pfam domain composition: all 51 proteins contained AA_permease, whereas 21 and 48 were additionally annotated with AA_permease_N and SLC12-related domains, respectively. (**d**) CDS exon–intron structures and genomic spans.

The conserved motifs, Pfam domains, and CDS exon–intron structures of the 51 SLC12 family members from the three tilapia species are shown in Figure 2. Motif arrangements were generally similar among members of the same orthologous group or subfamily, whereas motif number and organization differed among subfamilies. All 51 proteins contained an AA_permease domain; 48 were also annotated with an SLC12-related domain, and 21 with an AA_permease_N domain. The three SLC12A8 proteins contained the AA_permease domain but lacked detectable SLC12-related or AA_permease_N domains under the applied HMMER threshold. Exon number, intron length, and genomic span varied among members.

### 2.3. Chromosomal Distribution of SLC12 Genes in Three Tilapia Species

**Figure 3:**
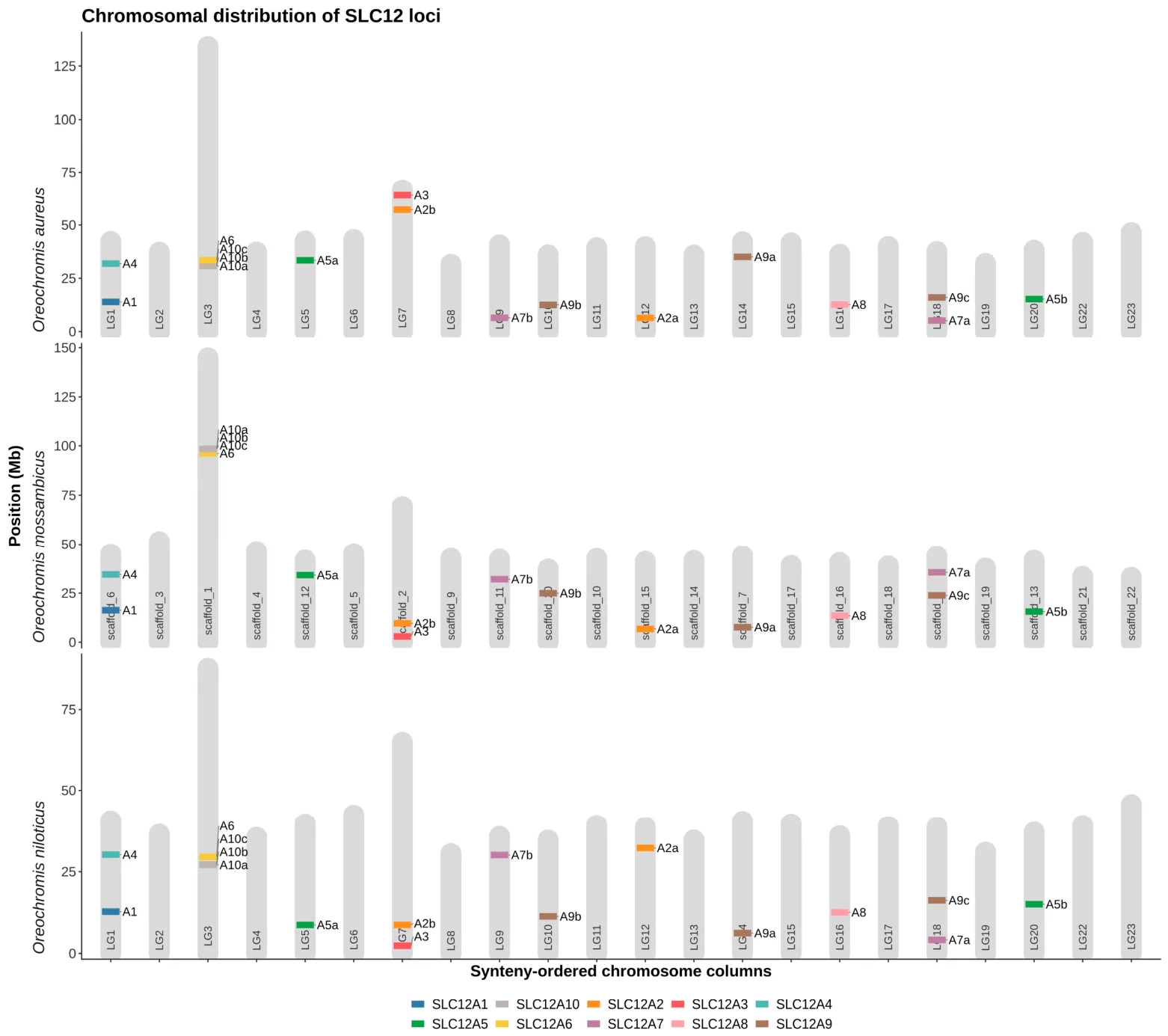
Chromosomal distribution of SLC12 genes in three tilapia species. The upper, middle, and lower panels represent blue tilapia (*Oreochromis aureus*), Mozambique tilapia (*O. mossambicus*), and Nile tilapia (*O. niloticus*), respectively. Different colors denote different SLC12 subfamilies. Chromosome columns were ordered according to interspecific syntenic relationships to facilitate comparison of the chromosomal correspondence of orthologous loci.

Chromosomal mapping showed that the 17 *SLC12* loci were distributed across 11 chromosome or scaffold groups in each of the three tilapia species (Figure 3). Nile tilapia and blue tilapia exhibited highly similar chromosomal distribution patterns. *OnSLC12A1* and *OnSLC12A4*, together with their blue tilapia orthologs *OaSLC12A1* and *OaSLC12A4*, were located on LG1. *OnSLC12A6* and *OnSLC12A10a–c*, as well as the corresponding *OaSLC12A6* and *OaSLC12A10a–c*, were located on LG3. Similarly, *On/OaSLC12A5a*, *SLC12A2b/SLC12A3*, *SLC12A7b*, *SLC12A9b*, *SLC12A2a*, *SLC12A9a*, *SLC12A8*, *SLC12A7a/SLC12A9c* and *SLC12A5b* were located on LG5, LG7, LG9, LG10, LG12, LG14, LG16, LG18, and LG20, respectively. Mozambique tilapia showed a comparable chromosomal distribution pattern for the corresponding OmSLC12 orthologs. When the chromosomes were arranged according to their homologous relationships with Nile and blue tilapia, the relative distribution of SLC12 loci was largely conserved among the three species. Notably, OnSLC12A7a and OnSLC12A9c are located on LG18, the same linkage group harboring the salinity-tolerance-associated QTL cluster identified in our previous studies, whereas OnSLC12A8 is located on LG16.

### 2.4. Tissue Expression Patterns of SLC12 Genes Under Long-Term Salinity Treatment

**Figure 4:**
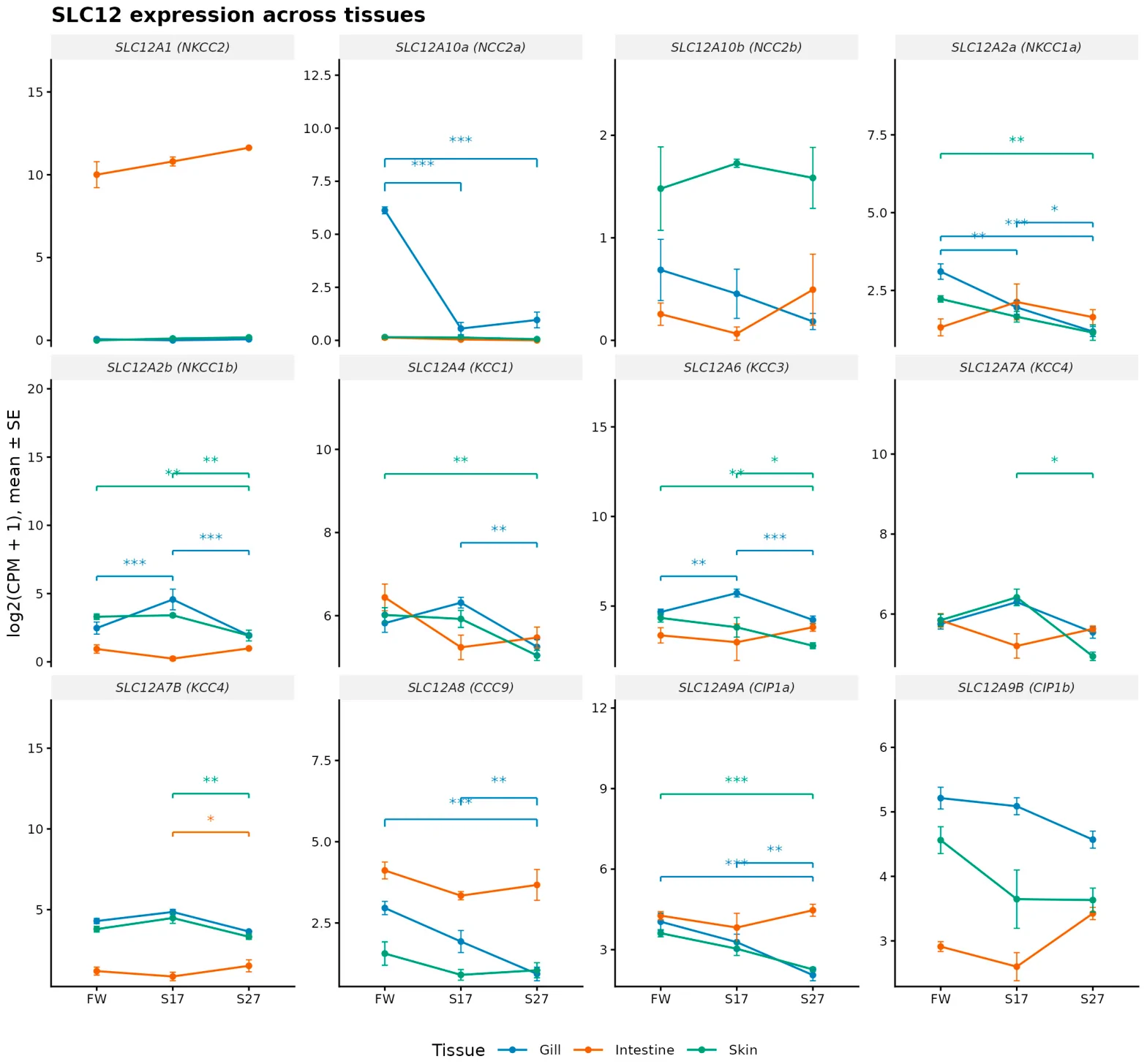
Tissue- and salinity-dependent expression patterns of SLC12 family members in GIFT Nile tilapia after 3.5 months of treatment. Treatments included freshwater (FW), salinity 17 (S17), and salinity 27 (S27), with four biological replicates for each tissue and salinity condition (*n* = 4). The *y*-axis shows log2 (CPM + 1), and data are presented as mean ± SE calculated from the four biological replicates. Differential expression was assessed by pairwise DESeq2 comparisons within each tissue. Horizontal brackets indicate the specific pairwise salinity comparisons. Significance annotations are shown only for comparisons with |log2FC| ≥ 1; *, padj < 0.05; **, padj < 0.01; ***, padj < 0.001.

In GIFT Nile tilapia, individual SLC12 members exhibited distinct tissue- and salinity-dependent expression patterns in the gill, intestine, and skin under long-term salinity treatment (Figure 4). Gill expression of *SLC12A10a* was highest under freshwater (FW) and markedly lower under salinity 17 (S17) and salinity 27 (S27), whereas *SLC12A10b* was expressed at a low overall level and varied only slightly among the three salinity conditions. *SLC12A2a* expression in the gill and skin generally decreased with increasing salinity, whereas gill *SLC12A2b* expression reached a higher level at S17 and declined at S27.

Among KCC members, *SLC12A4*, *SLC12A6*, *SLC12A7a*, and *SLC12A7b* exhibited distinct changes across tissues and salinity conditions. For example, gill *SLC12A6* expression reached a higher level at S17, whereas its expression in the skin decreased at S27; *SLC12A7a* expression in both gill and skin increased to a higher level at S17 and then declined.

*SLC12A8* expression was higher in the intestine than in the gill and skin. Its expression in the gill decreased progressively from FW to S27, whereas in the skin it decreased from FW to S17 and showed a slight increase at S27. *SLC12A9a* expression in the gill and skin decreased with increasing salinity, whereas its intestinal expression rebounded at S27. *SLC12A9b* was expressed at a relatively high level in the gill; in the skin, its expression decreased from FW to S17 and then remained at a similar level at S27.

OnSLC12A9c, one of the two SLC12 members located on LG18, showed consistently low expression across the examined tissues and salinity conditions and was therefore omitted from Figure 4 according to the visualization filtering criterion. In the gill, its expression showed an increasing trend at S17 relative to FW (log2FC = 2.111), but this difference was not statistically significant after multiple-testing correction (padj = 0.159) (Table S2).

### 2.5. Comparison of Ka, Ks, and Ka/Ks Across 17 Orthologous SLC12 Loci in Three Tilapia Species

The Ka, Ks, and Ka/Ks values obtained by comparing 17 *SLC12* orthologous loci from the three tilapia species with homologs from five external reference fish species showed broadly similar distributions (Figure S2). The Ka/Ks ranged from 0.023 to 0.279, with a mean of 0.120; all Ka/Ks values were below 1, indicating that the protein-coding sequences of the *SLC12* family were generally subject to strong purifying selection.

Mean Ka/Ks values varied among *SLC12* members. *SLC12A4* had the lowest mean Ka/Ks (0.043). Mean Ka/Ks values for *SLC12A5a*, *SLC12A5b*, and *SLC12A6* were 0.065, 0.069, and 0.077, respectively, indicating high sequence conservation. By comparison, the mean Ka/Ks of *SLC12A8* was 0.169; those of *SLC12A9a*, *SLC12A9b* and *SLC12A9c* were 0.152, 0.174 and 0.155, respectively; and those of *SLC12A10a*, *SLC12A10b* and *SLC12A10c* were 0.163, 0.128 and 0.151, respectively. Relatively higher mean Ka/Ks values were observed for *SLC12A9b* (0.174), *SLC12A8* (0.169), and *SLC12A10a* (0.163), although all values remained below 1. These comparisons were descriptive and were not used to infer statistically significant differences in evolutionary rate.

### 2.6. Sequence and Three-Dimensional Structural Comparison of SLC12A8 Among Three Tilapia Species

**Figure 5:**
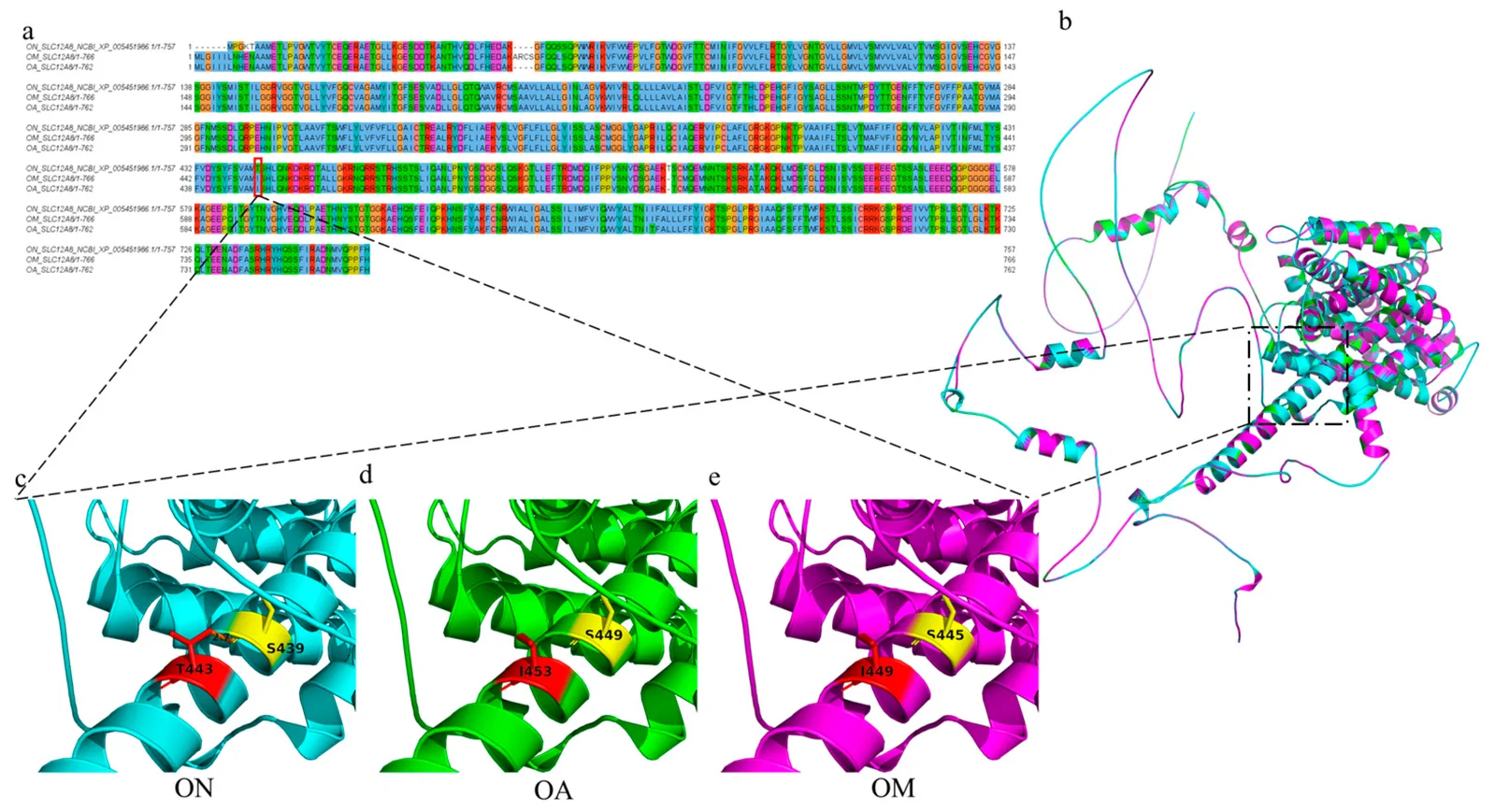
Sequence and predicted structural comparison of SLC12A8 among three tilapia species. (**a**) Multiple sequence alignment of OnSLC12A8, OmSLC12A8, and OaSLC12A8 and the homologous variable site. (**b**) Global superposition of the three predicted SLC12A8 structures. (**c**) Local structural environment surrounding OnSLC12A8 T443 and S439. (**d**) Local structure around OmSLC12A8 I453. (**e**) Local structure around OaSLC12A8 I449.

Multiple sequence alignment of SLC12A8 proteins showed that the overall sequences of OnSLC12A8, OmSLC12A8, and OaSLC12A8 were similar (Figure 5a). OnSLC12A8 contained Thr at position 443, whereas the homologous residues in OmSLC12A8 and OaSLC12A8 were Ile453 and Ile449, respectively. The SWISS-MODEL models for all three SLC12A8 proteins were constructed using the same template, A0A665XEB9, with GMQE values of approximately 0.66–0.67 and sequence identities to the template of approximately 79% (Figure 5b). Global superposition yielded RMSD values of approximately 0.37, 0.37, and 0.52 Å for the OmSLC12A8–OaSLC12A8, OaSLC12A8–OnSLC12A8, and OmSLC12A8–OnSLC12A8 comparisons, respectively. Because all three models were generated from the same template, these low RMSD values were interpreted only as descriptive measures of similarity within a common modeling framework rather than as independent evidence of structural conservation.

Local structural comparison showed that OnSLC12A8 T443, OmSLC12A8 I453, and OaSLC12A8 I449 occupied corresponding spatial positions in the predicted models (Figure 5c–e). In the OnSLC12A8 model, the polar side chain of T443 was positioned in proximity to the backbone carbonyl group of S439, suggesting a potentially distinct local physicochemical environment compared with the corresponding Ile-containing sites in OmSLC12A8 and OaSLC12A8. However, given the predictive nature of the homology models, no specific atomic interaction was inferred from this spatial proximity. Per-residue local model-quality scores for the five residues on either side of the homologous site ranged from 0.83 to 0.93, with T443, I449, and I453 each receiving a score of 0.87 (Table S3).

### 2.7. Comparison of SLC12A8 Transmembrane Topology Among Three Tilapia Species

**Figure 6:**
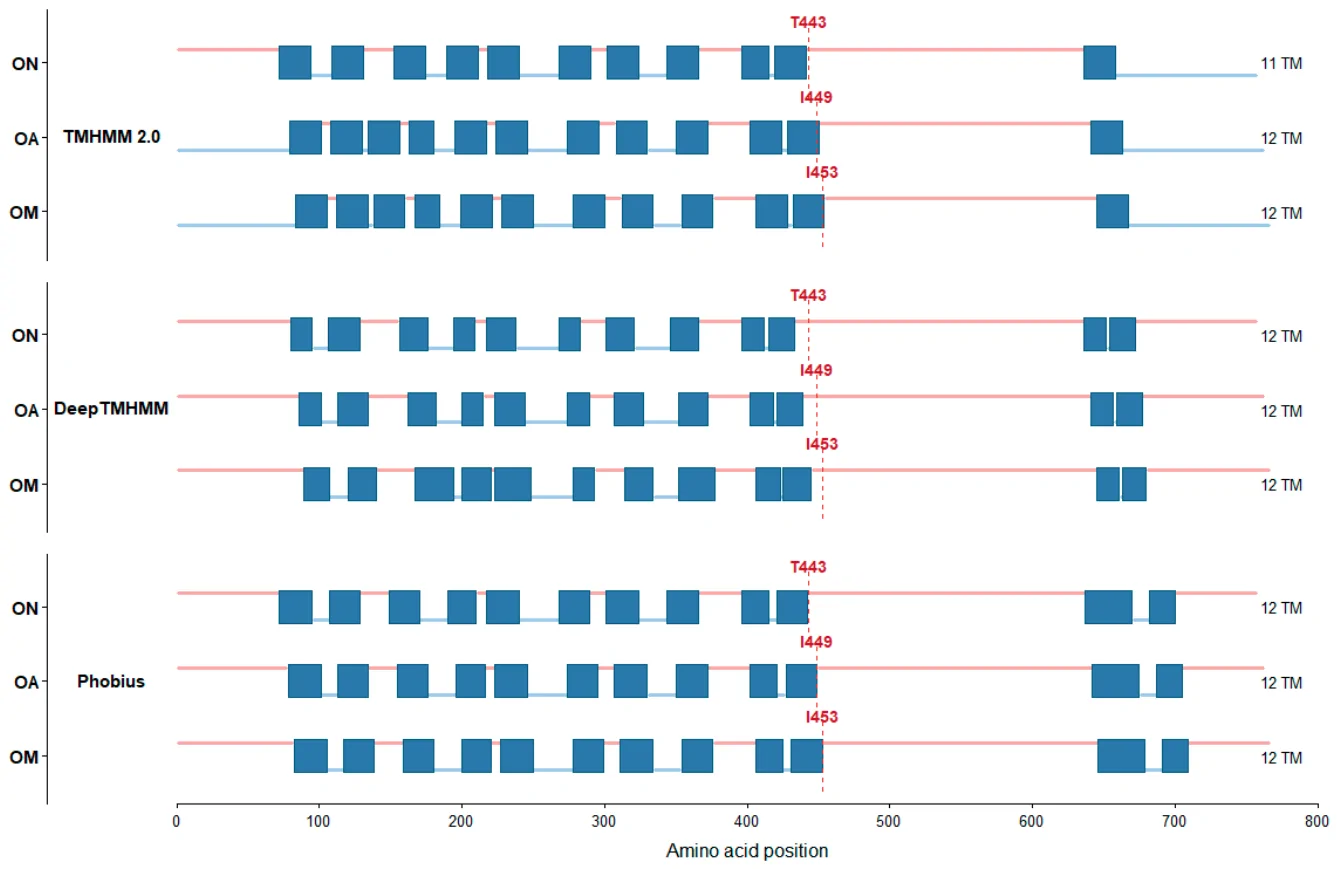
Cross-validation of SLC12A8 transmembrane-topology predictions among three tilapia species using TMHMM 2.0, DeepTMHMM, and Phobius. Predicted transmembrane helices are shown as blue rectangles, and dashed red lines indicate the homologous residues OnSLC12A8 T443, OaSLC12A8 I449, and OmSLC12A8 I453. TMHMM 2.0 predicted 11 transmembrane helices for OnSLC12A8 and 12 for OaSLC12A8 and OmSLC12A8, whereas DeepTMHMM and Phobius predicted 12 helices for all three proteins. The homologous T443/I449/I453 site was consistently located near the C-terminal boundary of the corresponding transmembrane helix, although the exact boundary varied among prediction methods.

Transmembrane topology of the three SLC12A8 orthologs was cross-validated using TMHMM 2.0, DeepTMHMM 1.0, and Phobius 1.01 (Figure 6). TMHMM 2.0 predicted 11 transmembrane helices for OnSLC12A8 and 12 for both OaSLC12A8 and OmSLC12A8, whereas DeepTMHMM and Phobius predicted 12 transmembrane helices for all three proteins. Thus, the 11-versus-12 difference was predictor-dependent and did not represent a consistently supported interspecific difference in overall transmembrane topology.

Notably, the homologous OnSLC12A8 T443, OaSLC12A8 I449, and OmSLC12A8 I453 residues were consistently located near the C-terminal boundary of the corresponding transmembrane helix. The precise position of this boundary varied among prediction methods: depending on the predictor and species, the homologous residue was located within the predicted helix, at its C-terminal end, or shortly downstream of the helix. These results indicate that the overall transmembrane architecture of SLC12A8 is broadly conserved among the three tilapia species, while the local membrane-boundary assignment surrounding the homologous T/I site is sensitive to the prediction method.

## 3. Discussion

### 3.1. The SLC12 Family Is Overall Conserved in Three Tilapia Species, and Ancient Gene Duplications Provide a Basis for Functional Divergence

We identified 17 SLC12 family members in each of Nile tilapia, Mozambique tilapia, and blue tilapia, with identical subfamily composition and copy numbers among the three species. SLC12A1, SLC12A3, SLC12A4, SLC12A6, and SLC12A8 were each present as a single copy; SLC12A2, SLC12A5, and SLC12A7 each retained two copies; and SLC12A9 and SLC12A10 each retained three copies. In addition, most orthologous members occupied corresponding chromosomal positions across the three species, and members of the same subfamily were generally similar in their conserved motifs and gene structures. These results indicate that, despite differences in salinity tolerance among the three tilapia species, the overall genomic composition of their SLC12 families remains highly conserved. Thus, differences in salinity adaptation among closely related *Oreochromis* species are unlikely to be explained simply by large-scale gains or losses of SLC12 members after species divergence and may instead involve more subtle differences in gene regulation or protein function that remain to be experimentally tested.

The widespread occurrence of paralogous genes in teleosts is closely related to their genomic evolutionary history. Early comparisons of zebrafish Hox gene clusters suggested that an additional genome duplication had occurred in the teleost ancestor, and subsequent studies of the pufferfish genome further supported the occurrence of whole-genome duplication in the teleost lineage, followed by extensive retention and loss of duplicated genes [14,15]. Following gene duplication, two copies may be maintained over long evolutionary periods through complementary degeneration of regulatory or functional elements, a process known as subfunctionalization. This mechanism provides an important theoretical basis for the retention of numerous paralogous genes in teleosts [16]. Phylogenetic analyses of the CCC/SLC12 family have likewise shown that this family underwent multiple rounds of gene duplication, lineage-specific loss, and post-duplication functional divergence during animal evolution. Thus, the present copy-number composition of different SLC12 members does not represent a simple recent expansion, but rather reflects the combined effects of a long evolutionary history [17].

Accordingly, the multicopy status of SLC12A2, SLC12A5, SLC12A7, SLC12A9, and SLC12A10 observed here is more likely to represent a legacy of ancient duplications shared by the three tilapia species. In particular, the identical numbers of duplicated copies and the well-conserved chromosomal correspondence among the three species suggest that the relevant duplication events likely predated the divergence of these *Oreochromis* species. From this perspective, gene duplication itself may not directly account for differences in salinity tolerance among the three tilapia species. Its evolutionary significance may instead lie in providing the genetic basis for the subsequent development of copy-specific differences in tissue expression and environmental responsiveness.

### 3.2. Differential Expression of Paralogous Members Suggests Post-Duplication Regulatory Divergence in the SLC12 Family

Under long-term salinity exposure, paralogous members within the same SLC12 subfamily did not exhibit identical salinity responses. For example, OnSLC12A2a expression in the gill and skin generally decreased with increasing salinity, whereas OnSLC12A2b expression in the gill reached a relatively high level at S17 and then declined at S27. OnSLC12A10a showed a clear pattern of high expression in the gill under freshwater conditions, whereas OnSLC12A10b was expressed at an overall low level and varied little among salinity conditions. Members such as OnSLC12A6 and OnSLC12A7a showed relatively pronounced expression peaks at the intermediate salinity. These expression differences are consistent with possible regulatory divergence among paralogous members.

This pattern is consistent with the complex transcriptional remodeling widely observed during salinity adaptation in euryhaline fishes. Comparisons of gill transcriptomes from freshwater- and seawater-acclimated Mozambique tilapia have shown that changes in salinity affect not only classical ion-transport genes, but also induce coordinated changes in energy metabolism, cellular remodeling, signal transduction, and multiple other processes [18]. Black-chinned tilapia likewise exhibit large-scale, salinity-specific transcriptional responses in the gill under different salinity conditions, and a recent study employing a broader salinity gradient further showed that gene expression does not generally follow a simple pattern of monotonic increase or decrease [19,20]. Hybrid tilapia also develop clearly distinct transcriptional regulatory networks under different osmotic stress conditions, further indicating that changes in the external osmotic environment elicit coordinated restructuring across multiple pathways rather than a linear change in a single ion-transport gene [21].

SLC12 members themselves also exhibit pronounced tissue and salinity dependence. In Mozambique tilapia, NCC, NKCC1a, and NHE3 are localized to different types of branchial mitochondrion-rich cells and show distinct changes in expression with environmental salinity, indicating that ion transporters can contribute to freshwater and seawater acclimation through the reorganization of ionocyte types and transporter expression levels [22]. Studies in Japanese eel have similarly found that gill NKCC1 expression increases with environmental salinity, whereas renal NCC is preferentially expressed under low-salinity conditions, demonstrating that the responses of SLC12 family members may differ markedly among organs [23,24].

Similar regulatory characteristics have also been reported in other euryhaline fishes. Branchial NKCC protein abundance increases significantly after Atlantic salmon enter seawater [25], whereas multiple ion-transport genes in euryhaline killifish exhibit clear time-dependent changes during rapid salinity transfer [26]. In alewife, acclimation to freshwater or seawater not only alters the levels of transporters such as NKCC1, but is also accompanied by remodeling of the composition and spatial distribution of branchial ionocytes [27]. More notably, two NCC homologs in sea lamprey display nearly complementary tissue- and salinity-dependent expression patterns: one is primarily involved in ion uptake by the gill and kidney under freshwater conditions, whereas the other is expressed mainly in the intestine under seawater conditions [28]. These findings indicate that the first change following gene duplication is not necessarily a complete shift in transported substrate; instead, it may involve a redistribution of tissue expression domains and environmental response conditions.

Therefore, the differing salinity responses among paralogous copies of *OnSLC12A2*, *OnSLC12A7* and *OnSLC12A10* observed in this study may reflect regulatory subfunctionalization following duplication. In particular, the increase in some members at S17 followed by a decline at S27 indicates that the long-term salinity response is not a simple relationship in which “the higher the salinity, the stronger the expression.” Different salinities may correspond to distinct, re-established osmotic steady states in which ionocyte composition, the direction of transepithelial transport, and the combinations of different transporters may all change. Rather than simply asking whether a particular gene is upregulated, determining the tissues and salinity ranges in which individual duplicate copies are preferentially recruited may more clearly reveal the functional divergence of the SLC12 family during long-term salinity adaptation. Beyond post-duplication regulatory divergence, these tissue- and salinity-dependent expression patterns may also reflect physiological plasticity during long-term salinity acclimation. Such plasticity may allow different SLC12 paralogs to be differentially recruited across tissues and salinity conditions in response to changes in osmotic demand.

### 3.3. SLC12A8 Exhibits Salinity-Responsive Expression and Interspecific Sequence Variation

Unlike *SLC12A2*, *SLC12A5*, *SLC12A7*, *SLC12A9* and *SLC12A10*, which have multiple paralogous copies, *SLC12A8* remained a single-copy gene in all three tilapia species. Comparisons with five external reference fishes showed that *SLC12A8* had similar mean Ka/Ks values among the three tilapia species, ranging from approximately 0.166 to 0.174. All values remained well below 1, consistent with overall purifying selection. Because the external reference species are not fully independent replicates, the small differences among the three tilapia species were treated as descriptive and were not interpreted as evidence of lineage-specific differences in evolutionary rate. It is against this conserved background that the small number of species-specific nonsynonymous substitutions merits further investigation.

From the perspective of its expression pattern, *OnSLC12A8* was expressed at a higher level in the intestine than in the gill or skin. Its expression in the gill decreased with increasing salinity, whereas in the skin it decreased from FW to S17 and showed a slight increase at S27. This pattern suggests that OnSLC12A8 may participate in transmembrane transport or cellular homeostatic processes related to long-term salinity adaptation, but the available evidence is insufficient to classify it directly as a canonical salt-ion transporter. In fact, compared with NKCC, NCC, and KCC, the substrate specificity and physiological function of SLC12A8 remain controversial. Daigle et al. [29] reported that human SLC12A8 could facilitate polyamine and amino acid transport, whereas Grozio et al. [30] subsequently proposed that it could mediate nicotinamide mononucleotide transport; the latter functional interpretation was subsequently challenged by other investigators [31]. It is therefore particularly inappropriate to infer the specific substrate transported by fish SLC12A8 directly from its mammalian annotation.

On the other hand, the fish intestine is itself an important organ for ion and water regulation during long-term adaptation to high salinity. For example, in euryhaline teleosts, intestinal NKCC2 can contribute to ion absorption and water balance during transitions to hyperosmotic environments [32]. This does not demonstrate that OnSLC12A8 performs the same function, but its relatively high intestinal expression raises the possibility that it may have physiological relevance in this tissue and warrants further functional investigation. Taken together, its single-copy status, strong purifying selection, tissue expression preference, and salinity responsiveness provide a reasonable basis for selecting SLC12A8 as a candidate for further sequence and structural comparisons in this study. Importantly, the salinity-responsive expression data and the interspecific sequence differences represent independent lines of evidence. Expression was examined only in GIFT Nile tilapia, and therefore the T443/I449/I453 variation among the three species cannot presently be associated with interspecific differences in expression or salinity tolerance. The sequence variation identified here should thus be regarded as a candidate feature for further investigation rather than as evidence of a causal contribution to species-specific salinity adaptation. Comparable expression and functional analyses across the three tilapia species will be required to establish such a relationship.

### 3.4. Local Polarity Changes in SLC12A8 May Be Associated with Structural Differences in a Transmembrane Region

Although the overall sequences and predicted three-dimensional structures of SLC12A8 were highly similar among the three tilapia species, we found that OnSLC12A8 contained Thr443, whereas the homologous residues in OmSLC12A8 and OaSLC12A8 were Ile453 and Ile449, respectively. Because all three homology models were generated using the same template, the low global RMSD values (approximately 0.37–0.52 Å) largely reflect the shared modeling framework and should not be regarded as independent evidence of structural conservation. Accordingly, these values are reported here only as descriptive measures of similarity among the predicted models. However, Ile is a hydrophobic, nonpolar residue, whereas Thr contains a polar hydroxyl group and differs substantially in polarity and hydropathy; thus, the I-to-T substitution is expected to alter the local physicochemical environment of this region [33,34]. In the OnSLC12A8 model, the polar side chain of T443 was positioned in proximity to the backbone carbonyl group of S439. This spatial arrangement suggests a potentially different local physicochemical environment from that surrounding the corresponding Ile residues in OaSLC12A8 and OmSLC12A8. However, the predictive nature of the homology models does not support inference of a specific hydrogen bond or other atomic interaction from this spatial proximity alone.

This local difference is structurally plausible. Cryo-electron microscopy structures of CCC family members, including NKCC1, KCC1, and KCC4, have shown that these proteins possess a highly conserved multipass transmembrane scaffold, but that ion binding, gating, and conformational transitions depend on a limited number of key residues within the transmembrane regions and on their local interactions [35,36,37,38]. Subsequent structures of KCC2, KCC3, and KCC proteins in different activation states further demonstrated that, even when the overall protein fold remains unchanged, alterations in local transmembrane-helix positions and side-chain interactions may still accompany pronounced differences in conformational state [39,40,41]. Because these studies were conducted in other vertebrate species, their relevance to tilapia SLC12A8 is inferred based on the general structural conservation of the CCC family [42].

Cross-validation with three transmembrane-topology predictors did not support a consistent interspecific difference in overall transmembrane-helix number. Although TMHMM 2.0 predicted 11 helices for OnSLC12A8 and 12 for OaSLC12A8 and OmSLC12A8, both DeepTMHMM and Phobius predicted 12 helices for all three proteins. The 11-versus-12 difference should therefore be regarded as predictor-dependent rather than as evidence that the I-to-T substitution causes the loss of a transmembrane helix.

Nevertheless, all three prediction methods placed the homologous T443/I449/I453 site near the C-terminal boundary of the corresponding transmembrane helix, although the exact boundary differed among algorithms. Thus, the I-to-T substitution occurs in a local membrane-boundary region whose precise topology is prediction-sensitive. This observation supports further examination of the site but does not establish a structural or functional consequence. Future studies could use I/T site-directed mutagenesis, subcellular localization, and substrate-transport assays to test its actual functional effects.

In summary, the SLC12 families of the three tilapia species display a clear pattern of “overall conservation with local divergence.” The three species share an identical family composition and relatively stable chromosomal correspondence, indicating that differences in salinity adaptation need not involve recent, large-scale changes in SLC12 gene number. Instead, the tissue- and salinity-responsive differences observed among multicopy members suggest that post-duplication regulatory divergence may enhance the flexibility of the SLC12 system in responding to environmental change. At the same time, the single-copy SLC12A8, while showing Ka/Ks values consistent with overall purifying selection, retains a species-specific local amino acid difference accompanied by a local change in residue polarity near a predictor-sensitive transmembrane boundary. Taken together, the present results reveal two distinct features of the SLC12 family: salinity-responsive transcriptional variation among SLC12 members in Nile tilapia and interspecific sequence variation in the conserved single-copy *SLC12A8*. These observations provide complementary candidates for future investigation, but their functional relationship and relevance to interspecific differences in salinity tolerance remain to be established.

## 4. Materials and Methods

### 4.1. Genome Data and Identification of Candidate SLC12 Genes

Reference genomes, protein sequences, and GFF/GTF annotation files for Nile tilapia (*Oreochromis niloticus*), blue tilapia (*O. aureus*), and Mozambique tilapia (*O. mossambicus*) were obtained from public databases, including NCBI, Ensembl, and Figshare (accessed on 30 July 2026). Chromosome-level genome assemblies were used for all three species. Specifically, the *O. niloticus* NCBI RefSeq assembly GCF_001858045.2, the O. aureus NCBI assembly GCF_013358895.1, and the chromosome-level South African PacBio HiFi/Omni-C assembly of *O. mossambicus*, together with their corresponding annotation files, were used for subsequent analyses. The *O. mossambicus* assembly was generated using PacBio HiFi and Omni-C sequencing, with 99.4% of the assembled sequences anchored to 22 chromosomes [43]. Some chromosome-scale sequences retain scaffold-style identifiers in the genome files; therefore, the scaffold labels reported in the Results refer to sequence identifiers rather than to the assembly level. The *O. mossambicus* genome and annotation files were obtained from Figshare dataset 30366010.

Reliably annotated SLC12 protein sequences from humans, zebrafish, and other representative teleosts were used as reference queries. Candidate SLC12 loci were initially retrieved by TBLASTN searches against the three tilapia genome assemblies with an E-value threshold of 1 × 10^−5^, and alignments with ≥30% amino-acid identity and ≥50% query coverage were retained for further evaluation. These relatively permissive criteria were used to minimize the risk of excluding divergent SLC12 homologs and were not used as the sole criteria for final gene identification. In parallel, HMMER searches of the annotated protein sets were performed using the SLC12-associated PF03522, PF00324 (AA_permease), and PF08403 (AA_permease_N) profile HMMs obtained from the Pfam database (accessed on 30 July 2026), with a domain E-value threshold of 1 × 10^−5^. Candidate loci supported by the TBLASTN homology search and Pfam-domain screening were combined and then evaluated by phylogenetic clustering with curated SLC12 reference proteins. The three Pfam profiles were treated as complementary evidence; simultaneous detection of all three profiles was not required, and no single Pfam hit was considered sufficient to establish SLC12 family membership. Final family membership was determined from the concordance of TBLASTN sequence similarity, Pfam-domain evidence, and placement within a recognized SLC12 phylogenetic clade. Transmembrane-topology analysis was performed after gene identification for structural characterization and was not used as a primary inclusion criterion. Sequence completeness was assessed from protein length and the presence of major N- and C-terminal truncations relative to full-length reference sequences; fragmentary or substantially truncated models and redundant isoforms were excluded. Within the resulting 51-protein set, all proteins contained PF00324 (AA_permease), whereas PF03522 (SLC12) and PF08403 (AA_permease_N) were detected in 48 and 21 proteins, respectively. These domain counts are reported descriptively and were not used as stand-alone evidence of family assignment. The three SLC12A8 proteins, which lacked detectable PF03522 and PF08403 under the applied threshold, were retained because both TBLASTN similarity and phylogenetic placement supported their assignment to the SLC12A8 clade. Conversely, related membrane transporters sharing an individual permease domain were excluded when they lacked concordant TBLASTN and phylogenetic support. Subfamily and paralog nomenclature was assigned according to clustering with curated SLC12 reference proteins in the phylogenetic tree.

### 4.2. Protein Physicochemical Properties and Transmembrane-Topology Prediction

Amino acid lengths were determined for representative SLC12 protein sequences from the three tilapia species. Molecular weight, theoretical isoelectric point (pI), instability index, and grand average of hydropathy (GRAVY) were calculated using the Bio.SeqUtils.ProtParam.ProteinAnalysis module in Biopython 1.87. For each locus, only the longest representative protein with a complete sequence and high annotation quality was selected for analysis to avoid duplicate counting caused by alternative splice isoforms.

In parallel, the TMHMM 2.0 online server was used to predict the number of transmembrane helices, transmembrane-region boundaries, and inside/outside membrane-topology orientations of SLC12 proteins. A total of 51 representative SLC12 protein sequences from the three tilapia species were included. For SLC12A8, transmembrane-topology predictions were additionally cross-validated using DeepTMHMM and Phobius. Predictions from the three methods were compared with particular attention to overall transmembrane-helix number and the position of the homologous T443/I449/I453 site relative to the C-terminal boundary of the corresponding transmembrane helix. Because the SLC12 family comprises typical multipass transmembrane transporters, the TMHMM predictions were used primarily to compare overall membrane-topology features among family members, assess the consistency of transmembrane-helix numbers, and help identify potentially anomalous or incomplete gene models.

### 4.3. Phylogenetic Analysis, Conserved Motifs, Domains, Gene Structure, and Chromosomal Distribution

SLC12 protein sequences from the three tilapia species were aligned with homologous sequences from representative vertebrates. Detailed information on the sequences used for phylogenetic analysis is provided in Table S1. Protein-sequence alignment was performed using MAFFT v7.526 with the parameters -auto -thread 24. A maximum-likelihood phylogenetic tree was constructed from the multiple-sequence alignment using IQ-TREE 2. ModelFinder selected Q.plant+R7 as the best-fitting model. Node support was evaluated using 1000 ultrafast bootstrap replicates and 1000 SH-aLRT replicates, with the parameters -bb 1000 -alrt 1000. The maximum-likelihood tree was visualized using Chiplot (https://www.chiplot.online/, accessed on 30 July 2026).

Conserved-motif analysis was performed on the 51 SLC12 protein sequences from the three tilapia species using MEME Suite v5.5.8 with the parameters -protein -nmotifs 15 -minw 6 -maxw 100 -mod zoops. MEME identified 15 motifs and ranked them according to statistical significance. For visualization, motifs 1–10, corresponding to the ten highest-ranked motifs with the lowest E-values, were displayed. Pfam-domain annotation was performed using the hmmscan program in HMMER v3.4, with primary searches against the SLC12-associated PF03522, PF00324 (AA_permease), and PF08403 (AA_permease_N) profile-HMM models (accessed on 30 July 2026). Domain positions and boundaries were determined from profile-HMM alignment coordinates.

The CDS, exon–intron structures, and chromosomal coordinates of each SLC12 gene were extracted from the GFF/GTF annotation files of the three species. Sequence and annotation files were read, CDS regions were extracted, the longest representative transcripts were selected, and genomic coordinates were compiled using Python 3. The main Biopython v1.87 modules used were Bio.SeqIO, Bio.AlignIO, Bio.Seq, and Bio.SeqUtils, together with the Python standard-library modules csv, re, and gzip for parsing annotation information and organizing data. When multiple alternatively spliced transcripts existed at the same locus, only the longest representative transcript with a complete sequence and high annotation quality was retained for subsequent analyses.

Phylogenetic relationships, gene structures, conserved motifs, and Pfam-domain information were integrated and visualized in R, primarily using the ape, ggplot2, patchwork, and xml2 packages.

### 4.4. Molecular Evolution and Ka/Ks Analysis

Codon-level molecular-evolution analyses were performed on orthologous SLC12 genes from the three tilapia species and their external reference sequences. Protein sequences were first aligned using MAFFT v7.526, after which PAL2NAL was used to generate codon alignments from the protein alignments and corresponding CDS sequences. The nonsynonymous substitution rate (Ka), synonymous substitution rate (Ks), and Ka/Ks ratio were calculated using KaKs_Calculator v3.0. The Nei–Gojobori (NG) model was used as the primary model, and the model-averaging (MA) model was used for supplementary comparison.

For each SLC12 family member, homologous sequences from zebrafish (*Danio rerio*), three-spined stickleback (*Gasterosteus aculeatus*), spotted gar (*Lepisosteus oculatus*), Japanese medaka (*Oryzias latipes*), and tiger pufferfish (*Takifugu rubripes*) were selected as external references. Ka, Ks, and Ka/Ks were calculated between each of the three tilapia species and each external reference to compare the strength of evolutionary constraints among SLC12 members. Ka/Ks < 1 was interpreted as evidence of overall purifying selection, and small differences in Ka/Ks among species were not directly interpreted as evidence of positive selection.

### 4.5. Transcriptomic Expression Analysis Under Long-Term Salinity Treatment

Published RNA-seq data from a long-term salinity treatment experiment in GIFT Nile tilapia were reanalyzed to characterize the expression patterns of SLC12 family members across tissues and salinity conditions. The experiment included freshwater (FW), salinity 17 (S17), and salinity 27 (S27) treatments. Gill, intestine, and skin tissues were collected after 3.5 months of treatment, with four biological replicates for each tissue and salinity condition, resulting in 36 paired-end libraries. The sequencing data were obtained from the DDBJ Sequence Read Archive under BioProject accessions PRJDB9175 and PRJDB10880. PRJDB9175 contained the skin transcriptome data, whereas PRJDB10880 contained the gill and intestine transcriptome data. All 36 libraries passed quality control and were retained for subsequent analyses [12,13].

Raw sequencing reads were processed using fastp v1.0.1. Clean paired-end reads were aligned to the Oreochromis niloticus reference genome assembly GCF_001858045.2 (O_niloticus_UMD_NMBU) using STAR v2.7.11b. Read counts for each gene were generated from coordinate-sorted BAM files using featureCounts v2.1.1 with paired-end fragment counting enabled (-p --countReadPairs). Reads were assigned to exon features and summarized according to the gene_id attribute using the unstranded option (-s 0). Gene annotation was based on O_niloticus_UMD_NMBU.115.

Differential expression analysis was performed using DESeq2 v1.46.0 on the original integer count matrix. Pairwise comparisons were conducted separately within each tissue for S17 versus FW, S27 versus FW, and S27 versus S17. Salinity was included as the experimental factor in the DESeq2 model. *p* values were adjusted using the Benjamini–Hochberg procedure for each comparison. Genes with padj < 0.05 and |log2FC| ≥ 1 were considered significantly differentially expressed. For visualization, SLC12 expression values were transformed as log2(CPM + 1). Genes with mean log2(CPM + 1) values below 1 across all nine tissue and salinity combinations were omitted from the main expression plot but retained in the complete expression matrix and differential expression analyses.

### 4.6. SLC12A8 Sequence Alignment, Three-Dimensional Structural Modeling, and Comparison of Local Residues

The OnSLC12A8, OmSLC12A8, and OaSLC12A8 protein sequences were first subjected to multiple-sequence alignment to identify homologous residue positions. Homology models were then constructed separately using SWISS-MODEL. The same template, A0A665XEB9 (from AlphaFold v2), was used for all three proteins. Model quality was evaluated using GMQE and local residue-quality scores. Based on the sequence correspondence, homologous Cα atoms were superposed by least squares, and global root-mean-square deviation (RMSD) values were calculated without iterative outlier rejection. The local spatial positions of OnSLC12A8 T443, OmSLC12A8 I453, and OaSLC12A8 I449 and their neighboring residues were compared qualitatively. Per-residue local model-quality scores for the five residues on either side of the homologous site were extracted from the B-factor field of the downloaded SWISS-MODEL PDB files. Because all three homology models were constructed using the same AlphaFold DB template (A0A665XEB9), these values were treated as template-derived local confidence estimates based on transferred pLDDT information rather than as independently calculated QMEANDisCo scores. No specific hydrogen bond or other atomic interaction was inferred from spatial proximity alone.

### 4.7. Statistical Analysis and Presentation of Results

The statistical significance of expression differences was determined from DESeq2-adjusted *p* values (padj). Cross-outgroup comparisons of Ka/Ks were used primarily to describe trends, and the different outgroup species were not treated as fully independent biological replicates. All structural differences were interpreted in conjunction with sequence alignments, model quality, and homologous positions to avoid inferring definitive functional changes solely from predicted models.

## Figures and Tables

**Table 1:** Comparison of SLC12 subfamily gene copy numbers among representative vertebrates.

Scientific Name	Code	Common Name	Total Loci	*A1* *NKCC2*	*A2* *NKCC1*	*A3* *NCC1*	*A4* *KCC1*	*A5* *KCC2*	*A6* *KCC3*	*A7* *KCC4*	*A8* *CCC9*	*A9* *CIP1/* *CCC6*	*A10* *NCC2*
*Homo sapiens*	HS	Human	9	1	1	1	1	1	1	1	1	1	0
*Xenopus tropicalis*	XT	Western clawed frog	10	1	1	1	1	1	1	0	1	2	1
*Lepisosteus oculatus*	LO	Spotted gar	10	1	1	1	1	1	0	1	1	2	1
*Danio rerio*	DR	Zebrafish	17	1	3	1	1	2	1	2	1	2	3
*Oryzias latipes*	OL	Japanese medaka	16	1	2	1	1	2	1	2	1	3	2
*Gasterosteus* *aculeatus*	GA	Three-spined stickleback	16	1	2	1	1	2	1	2	1	3	2
*Takifugu rubripes*	TR	Tiger puffer	14	1	2	1	1	1	1	2	1	4	0
*Leuciscus waleckii*	LW	Amur ide	17	2	2	1	1	2	1	3	0	2	3
*Oreochromis niloticus*	ON	Nile tilapia	17	1	2	1	1	2	1	2	1	3	3
*Oreochromis aureus*	OA	Blue tilapia	17	1	2	1	1	2	1	2	1	3	3
*Oreochromis* *mossambicus*	OM	Mozambique tilapia	17	1	2	1	1	2	1	2	1	3	3

**Table 2:** Physicochemical Properties of SLC12 Proteins in Three Tilapia Species.

Protein	Species	Length (aa)	Molecular Weight (Da)	Theoretical pI	Instability Index	GRAVY
OaSLC12A1	*Oreochromis aureus*	1042	115,394.41	7.75	35.13	0.1004
OaSLC12A2a	*Oreochromis aureus*	1096	119,999.80	6.49	39.25	0.1077
OaSLC12A2b	*Oreochromis aureus*	1151	125,641.22	5.75	34.17	−0.0056
OaSLC12A3	*Oreochromis aureus*	1028	112,840.54	7.66	42.42	0.0671
OaSLC12A4	*Oreochromis aureus*	1094	122,020.14	6.81	38.71	0.0580
OaSLC12A5a	*Oreochromis aureus*	1159	127,186.15	5.62	42.82	0.0196
OaSLC12A5b	*Oreochromis aureus*	1123	123,423.41	6.01	43.93	0.0619
OaSLC12A7a	*Oreochromis aureus*	1109	123,270.86	6.86	39.49	0.0770
OaSLC12A7b	*Oreochromis aureus*	1164	128,258.16	5.70	41.60	−0.0009
OaSLC12A8	*Oreochromis aureus*	762	83,183.92	6.17	40.01	0.2367
OaSLC12A9a	*Oreochromis aureus*	950	105,411.64	8.01	40.65	0.3087
OaSLC12A9b	*Oreochromis aureus*	930	101,915.75	6.44	39.74	0.4496
OaSLC12A9c	*Oreochromis aureus*	923	101,872.70	8.16	35.49	0.3731
OaSLC12A10a	*Oreochromis aureus*	1018	113,299.33	7.67	35.07	0.0441
OaSLC12A10b	*Oreochromis aureus*	1002	111,855.00	5.91	36.79	0.0470
OaSLC12A10c	*Oreochromis aureus*	1007	112,001.32	5.89	38.05	0.0198
OaSLC12A6	*Oreochromis aureus*	1187	130,773.09	6.57	35.44	0.0212
OmSLC12A1	*Oreochromis mossambicus*	1042	115,418.45	7.75	34.82	0.1006
OmSLC12A2a	*Oreochromis mossambicus*	1120	122,691.98	6.49	38.47	0.1424
OmSLC12A2b	*Oreochromis mossambicus*	1151	125,547.10	5.80	34.50	0.0010
OmSLC12A3	*Oreochromis mossambicus*	1028	112,937.79	7.87	42.37	0.0714
OmSLC12A4	*Oreochromis mossambicus*	1094	122,002.17	6.81	39.02	0.0625
OmSLC12A5a	*Oreochromis mossambicus*	1107	121,776.43	5.82	41.90	0.0653
OmSLC12A5b	*Oreochromis mossambicus*	1123	123,425.39	6.01	44.43	0.0577
OmSLC12A6	*Oreochromis mossambicus*	1212	133,655.22	6.55	35.86	0.0097
OmSLC12A7a	*Oreochromis mossambicus*	1109	123,216.76	6.60	39.24	0.0754
OmSLC12A7b	*Oreochromis mossambicus*	1164	128,302.21	5.70	41.77	−0.0010
OmSLC12A9a	*Oreochromis mossambicus*	950	105,411.64	8.01	40.65	0.3087
OmSLC12A9b	*Oreochromis mossambicus*	930	101,984.90	6.62	39.18	0.4511
OmSLC12A9c	*Oreochromis mossambicus*	923	101,774.58	8.14	35.23	0.3761
OmSLC12A10a	*Oreochromis mossambicus*	1020	113,634.75	6.53	36.40	0.0351
OmSLC12A10b	*Oreochromis mossambicus*	995	111,387.41	6.10	38.60	0.0178
OmSLC12A8	*Oreochromis mossambicus*	766	83,585.41	6.26	38.98	0.2402
OmSLC12A10c	*Oreochromis mossambicus*	1007	112,051.40	6.05	38.26	0.0175
OnSLC12A1	*Oreochromis niloticus*	1063	117,763.21	7.73	35.49	0.1021
OnSLC12A2a	*Oreochromis niloticus*	1157	126,333.00	7.09	37.02	0.1323
OnSLC12A2b	*Oreochromis niloticus*	1165	127,238.05	6.02	36.04	−0.0082
OnSLC12A3	*Oreochromis niloticus*	1028	112,871.56	7.66	42.18	0.0653
OnSLC12A4	*Oreochromis niloticus*	1094	121,970.02	6.81	39.00	0.0471
OnSLC12A5a	*Oreochromis niloticus*	1187	130,783.28	6.01	42.88	0.0105
OnSLC12A5b	*Oreochromis niloticus*	1123	123,393.39	6.08	44.10	0.0587
OnSLC12A6	*Oreochromis niloticus*	1187	130,773.09	6.57	35.44	0.0212
OnSLC12A7a	*Oreochromis niloticus*	1109	123,242.81	6.86	39.49	0.0748
OnSLC12A7b	*Oreochromis niloticus*	1181	130,792.32	6.20	41.19	−0.0342
OnSLC12A8	*Oreochromis niloticus*	757	82,553.11	6.33	40.65	0.2152
OnSLC12A9a	*Oreochromis niloticus*	950	105,411.64	8.01	40.65	0.3087
OnSLC12A9b	*Oreochromis niloticus*	903	98,862.22	6.82	39.69	0.4474
OnSLC12A9c	*Oreochromis niloticus*	921	101,588.31	8.16	34.70	0.3679
OnSLC12A10a	*Oreochromis niloticus*	994	110,680.47	6.73	35.66	0.0550
OnSLC12A10b	*Oreochromis niloticus*	1008	112,907.78	6.71	37.10	0.1116
OnSLC12A10c	*Oreochromis niloticus*	1056	117,579.75	6.19	39.10	0.0169

## Data Availability

The genomic datasets analyzed in this study are publicly available from NCBI, Ensembl, and Figshare, as described in the Materials and Methods. The RNA-seq datasets from long-term salinity-challenged GIFT Nile tilapia were obtained from previously published studies and are available in the DDBJ Sequence Read Archive under BioProject accession numbers PRJDB9175 and PRJDB10880.

## Supplementary Materials

The following supporting information can be downloaded at https://www.mdpi.com/article/10.3390/ijms27188124/s1.
