# Supplementary material for: Comparative Evolution of SLC12 Transporters in Tilapias and Salinity-Responsive Expression in Nile Tilapia
Source: Int J Mol Sci. 2026 Sep 12;27(18):8124. doi: 10.3390/ijms27188124 (PMC13607439; doi:10.3390/ijms27188124)
Supplement: Supplementary file 1 [file ijms-27-08124-s001.zip › Figure S2.pdf]

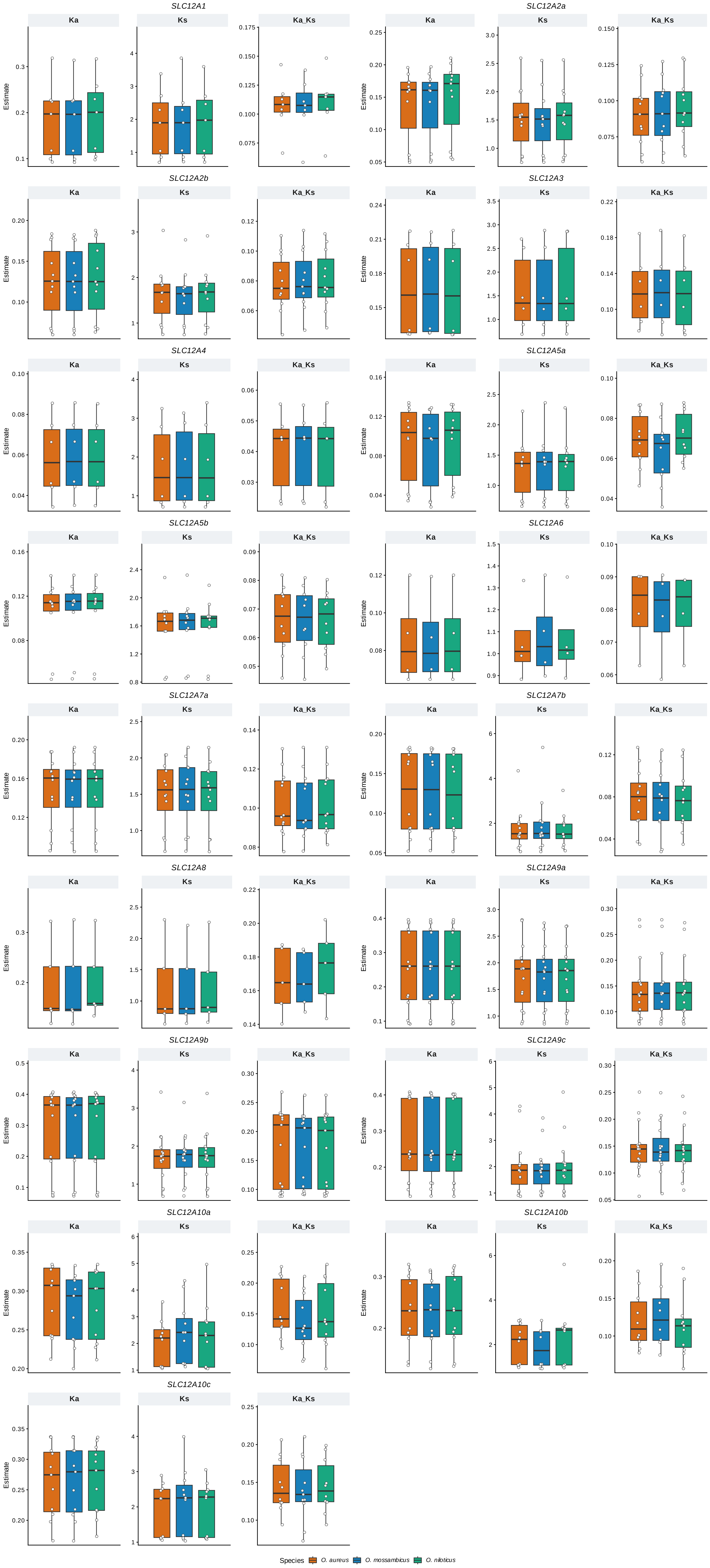
SLC12A1
SLC12A2a
SLC12A2b
SLC12A3
SLC12A4
SLC12A5a
SLC12A5b
SLC12A6
SLC12A7a
SLC12A7b
SLC12A8
SLC12A9a
SLC12A9b
SLC12A9c
SLC12A10a
SLC12A10b
SLC12A10c
Ka
Ks
Ka_Ks
Estimate
Species
O. aureus
O. mossambicus
O. niloticus
